# Role of Heterozygous APC Mutation in Niche Succession and Initiation of Colorectal Cancer – A Computational Study

**DOI:** 10.1371/journal.pone.0022720

**Published:** 2011-08-05

**Authors:** Roschen Sasikumar, John Raji Rejitha, Ponthananiyil Kumaran Binumon, Muraleedharan Manoj

**Affiliations:** Computational Modeling and Simulation Group, National Institute for Interdisciplinary Science and Technology (CSIR), Trivandrum, Kerala, India; Universität Heidelberg, Germany

## Abstract

Mutations in the adenomatous polyposis coli (APC) gene are found in most colorectal cancers. They cause constitutive activation of proliferative pathways when both alleles of the gene are mutated. However studies on individuals with familial adenomatous polyposis (FAP) have shown that a single mutated APC allele can also create changes in the precancerous colon crypt, like increased number of stem cells, increased crypt fission, greater variability of DNA methylation patterns, and higher somatic mutation rates. In this paper, using a computational model of colon crypt dynamics, we evolve and investigate a hypothesis on the effect of heterozygous APC mutation that explains these different observations. Based on previous reports and the results from the computational model we propose the hypothesis that heterozygous APC mutation has the effect of increasing the chances for a stem cell to divide symmetrically, producing two stem cell daughters. We incorporate this hypothesis into the model and perform simulation experiments to investigate the consequences of the hypothesis. Simulations show that this hypothesis links together the changes in FAP crypts observed in previous studies. The simulations also show that an APC^+/−^ stem cell gets selective advantages for dominating the crypt and progressing to cancer. This explains why most colon cancers are initiated by APC mutation. The results could have implications for preventing or retarding the onset of colon cancer in people with inherited or acquired mutation of one APC allele. Experimental validation of the hypothesis as well as investigation into the molecular mechanisms of this effect may therefore be worth undertaking.

## Introduction

Intestinal epithelium is a rapidly renewing tissue which renews itself every 4–6 days by a coordinated series of cell proliferation, migration and differentiation events [1]. These processes are compartmentalized in invaginations of the tissue called the crypts of Lieberkühn. A small population of stem cells, located at the base of the crypt within a niche is believed to divide continuously, producing semi differentiated transit cells. These semi-differentiated transit cells represent precursors at different stages of commitment and have the ability to divide rapidly a limited number of times, after which they undergo terminal differentiation. At the same time, as new cells are being produced, the entire population of semi- and terminally-differentiated cells migrates towards the luminal orifice where they are removed from the luminal surface.

Colorectal cancer arises as the cumulative effect of multiple mutations that enable the epithelial cell to escape all the controls that keep it from uncontrolled proliferation. Since in the colon mucosa, no cell other than the stem cells can survive more than a week, stem cells are the most reasonable candidates for the accumulation of multiple mutations. The initial genetic change in most colorectal adenomas is thought to be mutations in the tumor suppressor gene APC [2]. Mutations in APC can be identified in up to 80% of sporadic colorectal carcinomas. Individuals with heterozygous germline APC mutations as in Familial Adenomatous Polyposis (FAP) are born with normal appearing colons but hundreds of polyps start to appear during the second decade of life, suggesting that the normal APC allele also needs to become dysfunctional for the tumor to progress. However there are indications [3] that even the normal appearing FAP colon crypts may be harboring morphologically occult changes introduced by heterozygous APC mutation though the mechanism of these changes is not clear.

APC is a crucial member of the Wnt/β-catenin signaling pathway, which is an important determinant of cell proliferation, differentiation and apoptosis. APC also regulates cytoskeletal proteins including F-actin and microtubules, thus allowing it to regulate adhesion, migration and mitosis [2]. APC mutations generally result in truncated N-terminal protein fragments that cannot bind β-catenin and thus lose the function of Wnt/β-catenin regulation. Being a “loss of function” defect, heterozygous APC mutation is unlikely to have much effect on the Wnt/β-catenin signaling pathway. However it has been suggested that isolated N-terminal fragments can also have some “gain of function” effects on microtubules and spindle associated proteins in mitosis [4]. These effects can manifest even if only one APC allele is mutated.

Two types of division are possible for stem cells. In “asymmetric division” each stem cell generates exactly one stem cell and one semi differentiated (transit amplifying) cell at each division. The differentiated daughter cell leaves the niche to migrate up the crypt while the mother stem cell remains in the niche. In asymmetrical division, since the stem cells always replace themselves, their lineages never become extinct.

Stem cells can also divide “symmetrically”, producing either two semi differentiated daughters that leave the niche or two stem cell daughters that remain in the niche. Stem cells have the ability to switch between asymmetric and symmetric modes of division. The balance between these two modes of division is defective in some disease states [5]. Recent studies have shown that APC has a role in the process of regulating the balance between asymmetric and symmetric cell division by influencing the mitotic spindle orientation [6], [7].

Analysis of the variability of methylation patterns that arise in a crypt during aging provide evidence [8], [9] that normal human crypts get periodically taken over by descendants from one stem cell and this phenomenon is called “niche succession”. Unlike the clonal succession associated with tumor progression which occurs due to selection of a particular lineage that carries proliferative mutations, this is a random process unaided by selection. However it can provide a means by which tumor-initiating mutations can come to dominate the crypt well before tumor progression begins.

Niche succession is a consequence of symmetric stem cell division. With symmetric division, stem cell lineages become extinct whenever both daughters differentiate and leave the niche. To maintain a constant niche stem cell number, this extinction is balanced by expansion of another lineage by symmetric division in which both daughters remain as stem cells. This random stem cell loss with replacement can eventually lead to the extinction of all lineages except one, or “niche succession”. The intra-crypt variability of methylation tags is indicative of the period of niche succession, greater variability showing slower niche succession [3].

Methylation patterns in normal appearing FAP crypts show greater diversity than non FAP crypts indicating slower niche succession cycles in FAP crypts. This slowing down can be explained as due to an increase in stem cell population. FAP crypts also exhibit a shift in the distribution of proliferative cells along the crypt axis and this also has been linked to an increase in stem cell number [10]. Increased crypt fission observed in FAP colons [11] is also indicative of increase of stem cell number [12]. Therefore it has been suggested that heterozygous APC mutations may be contributing to increase in stem cell number [3].

In this paper we start with question, “What is the effect that a heterozygous APC mutation has on the behavior of an intestinal epithelial cell that can result in the changes observed in the precancerous FAP crypts?” The methodology we follow is that through computational experiments using an Agent Based model [13] of colon crypt dynamics we evolve a hypothesis on how the mutation changes cell behavior. We incorporate this hypothesis of individual cell behavior into the model and investigate the consequences of the hypothesis in the behavior of the crypt. Agent based modeling is a computational paradigm that is useful for investigating how individual behaviors lead to collective consequences. Agent based simulations monitor the actions and interactions of a large number of entities, or “agents”, in order to observe the aggregate behavior that emerges out of these individual actions. Each agent is described by its attributes and a set of rules that govern its behavior. Agents interact either directly with each other or indirectly through the environment and the emergent collective behavior is observed through simulations. The individual epithelial cells of the colon crypt are the agents in this model. Simulations reveal how assumptions of individual cell behavior lead to collective phenomena observable at the level of the crypt.

## Methods

The computer program that implements the model is developed using an ABM Framework developed in our group. The framework provides methods for defining agents and the rules that govern the actions they perform. A time loop is executed in which every agent and its environment is examined to see if the conditions for any action and consequent change of any attribute are fulfilled. The changes for all agents are effected together at the end of the time loop and the new time step starts with the changed conditions.

The framework is developed on VC.net platform.

### 2.1 Modeling Normal Crypt Dynamics

The normal crypt model is adapted from the model developed by Potten and Loeffler in 1987 [14]. The crypt is represented as a simple 2D grid of dimension N x M which would be as if the crypt is slit open and rolled out flat. The epithelial cells are anchored to the grid and can move on it. Each cell is an agent characterized by 7 attributes:

1. “State” (which specifies the Cell-cycle stage and can take values as “Quiescent” , “G1”, “S+G2”, “Mitosis” ),
2. “Position” (specified by the (x,y) co-ordinates on the grid),
3. “Time in State”(time spent in a particular state),
4. “Age” (time that has passed since birth),
5. “Number of Divisions” (number of times it has passed through mitosis),
6. “Stemness” (which defines the stage of differentiation/determination of the cell) and
7. “Ancestor” (the ancestral stem cell from which it has originated).

The basic model has nine parameters namely

1. Number of columns - N
2. Number of rows - M
3. Initial number of stem cells - N_0_
4. Cell Cycle Time for Stem cells - TC_s_,
5. Cell Cycle Time for Transit cells - TC_t_,
6. Time in G1 state by Stem cells - TG1_s_
7. Time in G1 state by Transit cells - TG1_t_
8. The maximum number of divisions before terminal differentiation - Num_div_max
9. Time step - Δt

#### 2.1.a. Initial Conditions

The bottom row of the grid is considered as the stem cell niche. Initially N_0_ stem cells are placed in the niche. All of them are initially assumed to be in G1 state but with different, randomly assigned values for the time that they have spent in that state. Therefore their division as well as the division of further generations is not synchronous. Time is incremented in steps of Δt.

#### 2.1.b. Rules for Division

- If State is ”Quiescent” and Stemness>0 state is changed to “G1”. Time in State is set to 0
- If state is “G1” and Time in State<TG1, Time in State is incremented by Δt
- If state is “G1” and Time in state > =  TG1 State is changed to “S+G2” and Time in State is set to 0
- If state is “S+G2” and Time in State< Cell cycle time –TG1, Time in State is incremented by Δt
- If state is “S+G2” and Time in state > =  Cell cycle time-TG1. State is changed to “Mitosis” and Time in State is set to 0
- If State is “Mitosis” Create and Insert daughter cell and set State of both to “Quiescent”

#### 2.1.c. Rules for Insertion of daughter cell

It is possible for more than one cell to occupy a grid space; however daughter cells are preferably inserted into empty neighboring grid. If there is no empty neighbor, it is possible to insert it into an occupied neighbor also. The priorities for choosing the insertion position in the descending order are given below

- Empty north neighbor
- Empty east or west neighbor ( random choice between them if both are empty)
- Empty south neighbor
- Occupied north , east or west neighbor (random choice)

Stem cell daughters are inserted always only into east or west neighbor to ensure that stem cells do not leave the niche

#### 2.1.d. Rules for Differentiation

With each division the progenies get more differentiated and become less stem-cell-like. Therefore the “Stemness” value decreases from 1 to 0 as the stem cells divide to form semi-differentiated progenitor cells (transit amplifying cells) that divide several times before becoming terminally differentiated cells that cannot divide any more. The maximum number of divisions a progenitor cell can undergo before full differentiation is fixed by an input parameter “Num_div_max”. The decrease in stemness per division is given by 1/ Num_div_max. After terminal differentiation the cells remain in state “Quiescent”.

#### 2.1.e. Rules for Migration

When new cells are born at the bottom, older cells get pushed up due to mitotic pressure. The model implements this through the rule that whenever more than one cell occupies the same grid space, the oldest cells are made to move up and push the whole column of cells above it by one grid space.

Stem cells never move out of the niche.

#### 2.1.f. Rule for Death / Shedding

Cells that reach the top row of the matrix are removed from the simulation to simulate cell shedding.

**Figure 1:**
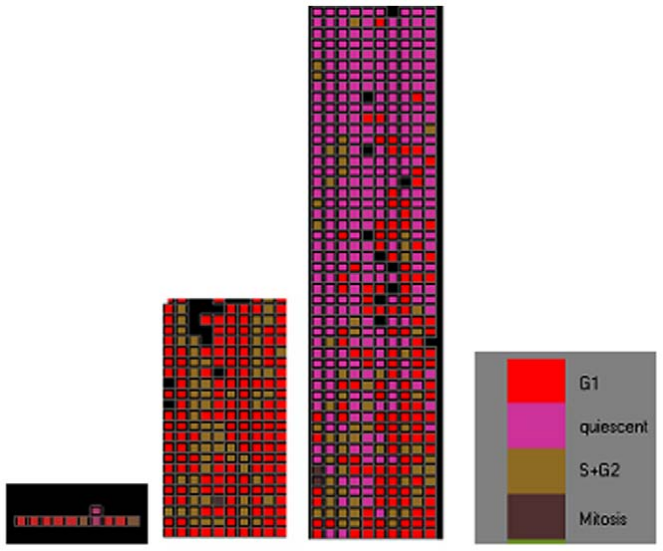
Development of a crypt from stem cells. States of the cells are represented by different colors : Purple – “Quiescent” , Red –“ G1”, Brown “S+G2”, Dark Brown – “Mitosis”, Black shows the gaps where there are no cells.

The input parameters used in the simulations are shown in Table 1. In order to save computation time only a portion of the colon crypt containing around 500 cells and a normal niche capacity of 10 stem cells is considered. A visualization of the development of a crypt from 10 stem cells using our model is given in Figure 1. While the absolute values of the observables like the number of stem cells or niche succession period that we determine from this model would not be valid for the whole crypt, it is assumed that the ***variations*** of the observables that we get out of the simulations are representative of the trends of behavior of these observables in the crypt. Since our interest is to evaluate the changes that APC mutation would make on the crypt it is sufficient to obtain the trends of behavior rather than the absolute values. The values pertaining to cell division behavior are taken from References [15] and [16].

**Table 1:** Parameter Values.

Parameter	Value
Number of columns - N	10
Number of rows - M	50
Initial number of stem cells - N_0_	10
Cell Cycle Time for Stem cells - TC_s_	24 hours
Cell Cycle Time for Transit Amplifying cells - TC_t_	12 hours
Time in G1 state by Stem cells TG1_s_	12 hours
Time in G1 state by Transit Amplifying cells – TG1_t_	4 hours
The maximum number of divisions before terminal differentiation – Num_div_max	6
Time step Δt	30 minutes

### 2.2 Modeling Symmetric Stem cell division and Niche Succession

In the basic crypt model, stem cell division is considered to be purely asymmetric, each division resulting in one stem cell that remains in the niche and one differentiated cell which is capable of leaving the niche. We include the possibility of symmetric division as follows:

A parameter **“symmetric division probability (P_s_)”** is defined as input. This parameter specifies the probability that a stem cell division is symmetric. Whenever a stem cell divides we generate a random number between 0 and 1. If the probability of symmetric division is greater than the random number the division is deemed symmetric. Symmetric division can be of two types with both daughters having stemness = 1 or with both daughters equally differentiated. An additional parameter “**differentiation probability** (P_d_)” decides whether the progeny will be two differentiated cells or two stem cells.

The probabilities that a cell division leads to 0, 1 or 2 stem cells are related to P_s_ and P_d_ as:



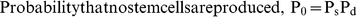
(1)




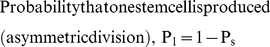
(2)




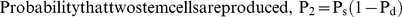
(3)


For normal crypts the stem cell number needs to be maintained constant which is possible only if there are equal chances for both types of symmetric division. This implies that P_0_ must be equal to P_2_ and therefore the differentiation probability P_d_ should be 0.5. However in practice we found that setting the differentiation probability to 0.5 was not sufficient to maintain the stem cell number in our stochastic simulations. To ensure stability of the stochastic numerical computations we introduce a correction factor to the differentiation probability that corrects for deviations of the actual stem cell number N_s_ from the original stem cell number N_0_.



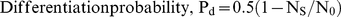
(4)


The correction factor (1-N_s_/N_0_ ) ensures that when the number of stem cells goes above N_0_ the differentiation probability increases above 0.5 producing more differentiated cells than stem cells and vice versa.

We assume that in abnormal colon crypts the stem cell number may not be maintained constant. And therefore in our model P_0_ and P_2_ can be different unlike in existing models where P_0_ and P_2_ are kept equal. A biasing factor B is introduced which represents a bias in the symmetric division towards production of stem cell progeny. The probability of differentiation during a symmetric division is calculated by multiplying the corrected differentiation probability by the biasing factor , B. When B<1, the probability of stem cell progeny increases and vice versa.

Starting with N_0_ stem cells, the time taken for all the stem cells to become descendants of one of the initial stem cells is taken as the niche succession period.

### 2.3 Modeling the effects of Heterozygous APC Mutation

Our objective is to evolve and test through computational experiments, a hypothesis on the difference made by a heterozygous mutation of APC to the individual cell behavior. The hypothesis is tested by seeing whether with this assumption on individual cell behavior, the observed changes in FAP crypts are shown up in the simulations.

Every cell agent is assumed to possess two copies of the APC gene which can be in a mutated or un-mutated state. The mutation state of a gene is represented as an attribute of the cell agent which can take values “0” or “1” for un-mutated or mutated state respectively. For modeling FAP all the stem cells are assumed to have one mutated APC gene. For modeling sporadic cancer the simulation begins at a point when one of the stem cells has acquired a mutation in one of the APC alleles. Somatic mutation of the second APC allele is assumed to take place with a probability defined by an input parameter **“Mutation probability”** which defines the probability that the gene gets mutated during the division of a cell. When the mutation probability is greater than a random number generated during division of the cell, the gene is assumed to become mutated and its mutation state is set to “1”. A cell with a mutated APC gene is assumed to acquire a change in the symmetric division probability or differentiation probability or both.

## Results and Discussion

The computational experiments described here are aimed at evolving and supporting a hypothesis on the effect of heterozygous APC mutation on individual cell behavior such that this behavior would collectively lead to the kind of crypt level changes that have been indicated by different experimental studies.

### 3.1 Effect of changes in Symmetric Division Probability on the Niche Succession Period in FAP crypts

The first set of experiments determines how the niche succession period is affected by changes in the probability of symmetric division.

We start with 10 stem cells of different lineages SC0 to SC9. The corrected differentiation probability (Equation (4) of Section 2.2) maintains the number of stem cells mostly in the 8–13 range with an average value of 10. The time taken for all the stem cells to become descendants of one of the initial stem cells is the niche succession period. Niche succession results in all the non-stem cells in the crypt also becoming descendants of the dominant stem cell lineage that succeeds in capturing the niche.

**Figure 2:**
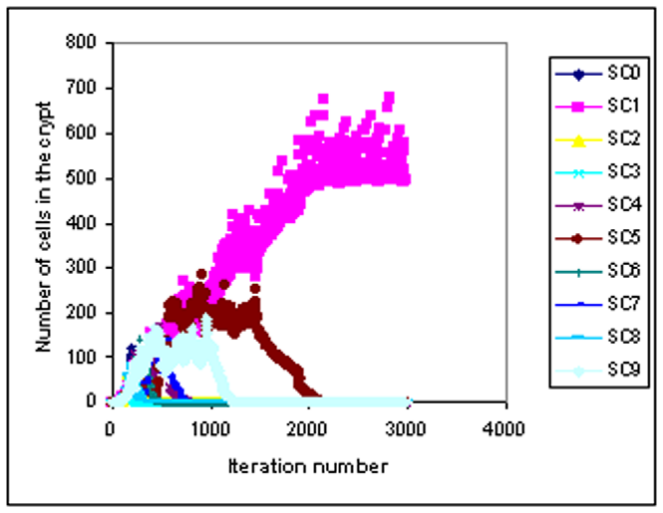
Variation of the total number of cells from each stem cell lineage with time. Stem cell number and Niche Succession Period for different values of symmetric division probability and the biasing factor.

Figure 2 shows an example of how the stem cell lineages become extinct one by one and finally descendants of one stem cell take over the whole crypt. It can be noted in Figure 2 that after around 2200 iterations only the lineage from the stem cell SC1 survives indicating that this lineage has taken over the niche.

**Figure 3:**
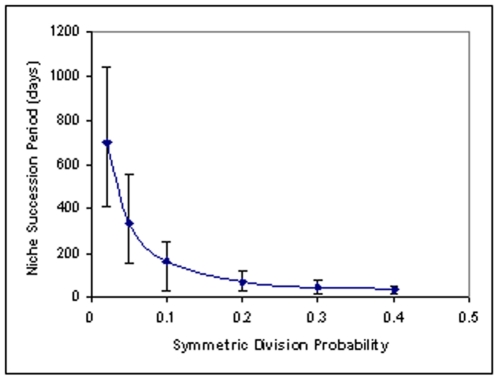
Variation of niche succession period with symmetric division probability. Probability of niche succession assuming different effects for the APC+/− mutation.

Figure 3 shows the variation of the average niche succession period with the symmetric division probability Ps. The niche succession period is seen to decrease with increase of Ps, the initial steep change flattening as the symmetric division probability increases. For pure asymmetric division (Ps = 0) there can be no extinction of any stem cell lineage and therefore the niche succession period tends to infinity as Ps tends to zero. As Ps increases stem cell lineages acquire a finite probability for becoming extinct by symmetric differentiation and therefore the period of niche succession decreases. As Ps increases the probability of survival of a lineage by symmetric production of stem cell progeny also increases along with the probability for extinction by symmetric differentiation. Therefore the slope of the curve decreases with increase of Ps. Similar trend of behavior has been obtained by van Leeuwen et al. [17]

### 3.2 Evolving a hypothesis on the effect of APC Mutation

There is an apparent contradiction between the results of Figure 3 and experimental observations:

- Figure 3 shows that niche succession period decreases when symmetric division increases.
- Methylation pattern analysis shows that niche succession period increases in APC mutated (FAP) crypts [3]. This implies that symmetric division probability is reduced in FAP crypts as per Figure 3.
- However it has been reported [6], [7] that APC mutation results in loss of asymmetric division in stem cells i.e. symmetric division probability increases in APC mutant stem cells.

We attempt to resolve this contradiction by hypothesizing that APC mutation not only increases symmetric division, but also biases the division in favor of producing stem cell progeny. In the normal crypt it is assumed that there are equal chances for differentiated progeny and stem cell progeny in symmetric division. If, in addition to increasing symmetric division, the APC mutation biases the symmetric division in favor of stem cell progeny, the stem cell number would increase and it is likely that the larger number of stem cells would result in increasing the niche succession period.

#### 3.2.a Effect of biasing symmetric division in favor of stem cell progeny

The maintenance of stem cell number must be having an environmental control mechanism that gives global signals as to whether stem cell number needs to be increased or decreased. APC mutation may not have an impact on this environmental control while the response of the cell to the environmental control may be affected by the mutation. The corrected differentiation probability (Equation 4) that attempts to keep the stem cell number constant can be considered as a computational representation of the environmental control. The effect of APC mutation in biasing the division in favor of stem cell progeny is then represented by reducing the corrected differentiation probability by multiplying it by a biasing factor less than 1. The corrected differentiation probability calculated by Equation 4 can take values in the range 0 to 1 depending on the number of stem cells at any point of time. Values of differentiation probability less than zero corresponding to N_s_/N_0_<0.5 are restricted to zero and values greater than one corresponding to N_s_/N_0_ >1.5 are restricted to one. With no bias (B = 1) the probability of producing two stem cells P_2_ and the probability of producing no stem cells P_0_ are in the same range. The ranges of values possible for P_0_ and P_2_ for different bias factors are shown in Table 2. When the biasing factor is less than 1 it becomes possible for P_2_ to have values greater than P_0_ . For example for Ps = .2 and B = 0.6, the biased differentiation probability can vary between 0 and 0.6 and P_0_ can vary between 0 and 0.12 and P_2_ can vary between 0.08 and 0.2. P_0_ and P_2_ have an overlapping range (.08 to 0.12 ) outside of which P_2_ takes values higher than P_0_. When the biasing factor is less than 0.5 P_2_ remains constantly larger than P_0_.

**Table 2:** 

Symmetric division probability P_s_	Biasing factor for differentiation probability B	Range of biased differentiation probability (P_d_)	Range of probability of producing 0 stem cells P_0_ = P_s_ P_d_	Range of probability of producing 2 stem cells P_2_ = P_s_(1-P_d_)	Average stem cell number	Range of stem cell number (between 5^th^ and 95^th^ percentile)	Average Niche succession period (days)
0.2	1	0 – 1	0 – .2	0 – .2	10	8–13	60
	0.8	0 –.8	0 –.16	.04 – .2	11	9–14	66
	0.7	0 –.7	0 –.14	.06 – .2	12	10–16	78
	0.6	0 – .6	0 – .12	.08 – .2	13	10–18	70
	0.5	0 –.5	0 –.1	.1 – .2	15	11–20	99
	0.4	0 –.4	0 –.08 (always lower than P_2_ )	.12 – .2 (always higher than P_0_ )	Continuous increase probably leading to crypt fission	∼	More than one lineage survives
0.1	1	0 – 1	0 – .1	0 – .1	10	8–13	100
	0.8	0 –.8	0 –.08	.02 – .1	11	9–14	120
	0.7	0 –.7	0 –.07	.03 – .1	12	11–16	156
	0.6	0 – .6	0 – .06	.04 – .1	13	11–18	215
	0.5	0 –.5	0 –.05	.05 – .1	32	13–83	374
	0.4	0 –.4	0 –.04 (always lower than P_2_ )	.06 – .1 (always higher than P_0_ )	Continuous increase probably leading to crypt fission	∼	More than one lineage survives
0.05	1	0 – 1	0 – .05	0 – .05	10	8–13	339
	0.8	0 –.8	0 –.04	.01 – .05	11	9–15	376
	0.7	0 –.7	0 –.035	.015 – .05	12	9–16	428
	0.6	0 – .6	0 – .03	.02 – .05	13	12–20	458
	0.5	0 –.5	0 –.025	.025 – .05	55	14–132	862
	0.4	0 –.4	0 –.02 (always lower than P_2_ )	.03 – .05 (always higher than P_0_ )	Continuous increase probably leading to crypt fission	∼	More than one lineage survives
0.02	1	0 – 1	0 – .02	0 – .02	10	7–13	697
	0.8	0 –.8	0 –.016	.004 – .02	11	8–14	829
	0.7	0 –.7	0 –.014	.006 – .02	12	7–15	920
	0.6	0 – .6	0 – .12	.008 – .02	13	9 –17	1234
	0.5	0 –.5	0 –.01	.01 – .02	31	13–68	2339
	0.4	0 –.4	0 –.008 (always lower than P_2_ )	.012 – .02 (always higher than P_0_ )	Continuous increase probably leading to crypt fission	∼	More than one lineage survives

**Figure 4:**
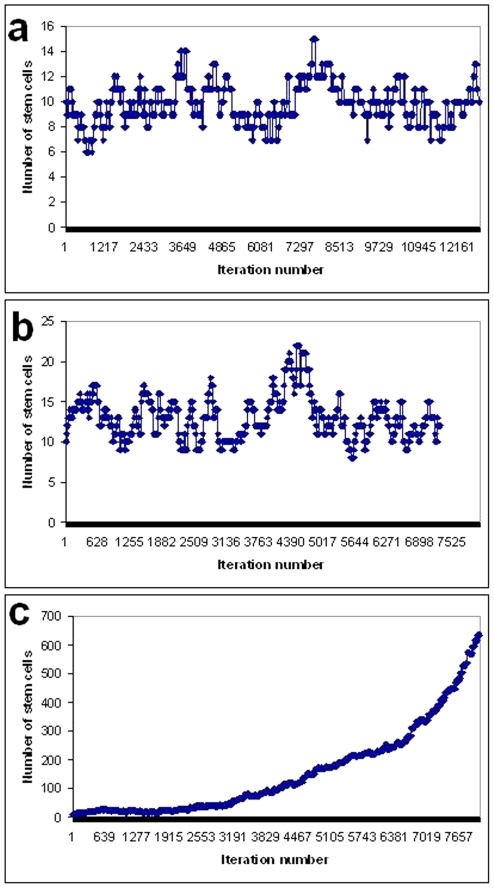
Time variation of stem cell number with: (a) Bias factor  = 1, (b) Bias factor  = 0.5, (c) Bias factor  = 0.4. Time taken for the appearance of a second mutation assuming different effects for the APC+/− mutation.

Figures 4 a–c shows the variation of stem cell number with time (iteration number) for different values of the biasing factor. When the biasing factor equals 1 and P_0_ and P_2_ are in the same range the number of stem cells is maintained within a range 8–13 with an average value of 10 (Figure 4 a). For values of the biasing factor between 1 and 0.5, The ranges of P_2_ and P_0_ are such that P_2_ can assume values greater than P_0_ and therefore the average stem cell number increases but the correction factor still controls the stem cell number from inordinate increase (Figure 4 b). When the biasing factor is made less than 0.5 the stem cell number increases uncontrollably (Figure 4 c) because P_2_ is always larger than P_0_.

Table 2 shows the stem cell number and niche succession period for different values of symmetric division probability and the biasing factor. Change of symmetric division probability is seen to have no effect on the stem cell number as long as there is no bias in the differentiation probability. The niche succession period is affected by both symmetric division probability and differentiation probability. For the same differentiation probability the niche succession period decreases as the symmetric division probability increases. For the same symmetric division probability the niche succession period increases as the biasing factor decreases. If the differentiation probability decreases along with increase of symmetric division probability the niche succession period can increase in certain cases. For example when symmetric division probability increases from 0.02 to 0.05 and at the same time the biasing factor for differentiation probability decreases from 1 to 0.5 the niche succession period increases from 697 days to 812 days. However on the whole the effect of the biasing factor is not very dramatic as long as it is above 0.5. When the biasing factor goes below 0.5 the simulations show uncontrolled increase of stem cell number. This would correspond to a situation where the mutated stem cells completely ignore the environmental signal to produce differentiated progeny. The large number of stem cells ensures that two or three lineages continue to persist and so complete niche take over by a single lineage gets delayed indefinitely. In fact we found that computer memory overflow occurs before niche succession could be observed. In real crypts it would be impossible for the niche to contain such large number of stem cells and probably the pressure of overcrowding would lead to crypt fission which is not dealt with in this model.

Thus the hypothesis that heterozygous APC mutation not only increases symmetric division probability but also biases symmetric division towards producing stem cell progeny, is consistent with the following observations from previous studies:

- Heterozygous APC mutation increases symmetric division in stem cells [6], [7]
- Precancerous FAP crypts show increased niche succession period [3]
- Stem cell number is increased in FAP crypts [3], [10]–[12]

On the other hand if APC mutation only increases symmetric division, the niche succession period would decrease and there would be no increase in stem cell number. Therefore the bias in favor of stem cell progeny is essential to explain the observed changes in FAP crypts.

The molecular mechanism of how heterozygous APC mutation increases and biases symmetric division is uncertain. Anchoring of stem cells in the niche appears to play an important role in the decision to divide symmetrically or asymmetrically [7], [8], [18] as well as the decision to differentiate or not [19], [20]. Whether the anchoring is related to APC control of the WNT pathway or whether it is through some other mechanism , has to be investigated. It has been suggested that haploinsufficiency in APC's control of the WNT pathway [3] results in accumulation of β-Catenin which in turn affects adherens junctions between the stem cells and the niche and influences the cell's decision to differentiate or not [19], [20]. Another possibility is the effect of N-terminal fragments of mutated APC on microtubules and spindle associated proteins in mitosis [4].

In the following sections we show that if heterozygous APC mutation has the effect of increasing as well as biasing symmetric division, cells with this mutation have a good possibility of initiating colorectal cancer.

### 3.3 Sporadic mutation that increases biased symmetric division has a good chance of getting fixed in the crypt

Previous theoretical studies indicate that symmetric division (and consequent niche succession) protects the cell from accumulation of mutations [17], [21] because a stem cell lineage bearing one mutation “has more chance of becoming extinct during the next niche succession cycle than of benefiting from the advantages of fixation in the crypt”. However these results are valid only if the mutation is neutral from the point of view of niche succession. If, on the other hand, the mutation has the effect of giving a selective advantage for niche succession, the mutation would be able to “hitch-hike” on niche succession and get fixed in the crypt.

The observation that most sporadic colon cancers are initiated by APC mutation suggests that the first APC mutation has a better chance than any other mutation for getting fixed in the crypt by niche succession. We show below that if the effect of heterozygous APC mutation is only to increase symmetric division, an APC mutated stem cell lineage has a higher chance of getting extinct. On the other hand if the effect of the mutation is not only to increase symmetric division, but also to bias it in favor of stem cell progeny, we show below that the mutation has a high probability of getting fixed in the crypt.

We performed simulations where 9 out of the 10 stem cells were considered normal (control) cells, and one stem cell was assumed to have a mutation that makes its symmetric division probability twice the control probability and a differentiation probability that is biased to different degrees in favor of producing stem cell progeny. The control symmetric division probability is assumed to be 0.1. The results are shown in Table 3. A mutation that enhances biased symmetric division increases the probability of fixation significantly above the random (one in ten) probability of 10%, the probability increasing as the division gets more and more biased in favor of stem cell progeny. On the other hand increase of symmetric division without the bias decreases the probability of fixation to below the random probability.

**Table 3:** 

Effect of mutation	Probability of niche succession by the mutated cell
No effect	10%
Symmetric division probability is doubled but unbiased	3%
Symmetric division probability is doubled and biased B = 0.6	45%
Symmetric division probability is doubled and biased B = 0.5	75%
Rate of division is doubled	0%

Interestingly, when it was assumed that the effect of the mutation is to increase the rate of division by a factor of 2 the mutated cell lineage was lost in all the simulations because the increased number of divisions only increases the chances of its extinction. Therefore a proliferative advantage actually seems to work against the survival of the stem cell lineage. It is thus likely that if our hypothesis is true, a sporadic heterozygous APC mutation gets fixed in the crypt before the acquisition of proliferative mutations. This fits very well with the observation that most sporadic colorectal cancers are initiated by APC mutation even though there are other mutations like in KRAS or β-Catenin that could as well enhance cell proliferation.

### 3.4 Mutation that increases biased symmetric division increases somatic mutation rates

In FAP colons the onset of cancer is faster than what could be expected if the pre-existing APC mutation implies only that the cells need one mutation less in order to initiate cancer [21]. Non-neoplastic FAP crypts are reported to have higher mutation rates [22], [23] which implies that heterozygous APC mutation has the effect of increasing mutation rates.

An interesting computational study by Pepper etal [24] shows that serial differentiation is the key to suppression of somatic mutation in multicellular tissues. Asymmetric division as well as self-renewing symmetric division increase somatic mutation rate in a tissue by increasing the number of cells in the proliferating pool. In our model transit amplifying cells undergo serial differentiation by always dividing symmetrically into further differentiated progeny. The stem cells on the other hand have the capability for all the three modes of division viz. asymmetric, self-renewing symmetric and differentiated symmetric. Table 4 shows the iteration number at which the somatic mutation of the second APC allele takes place for different values of symmetric division probability and differentiation probability. The mutation probability of the normal APC allele is assumed to be .001 and normal symmetric division probability is set to 0.1. APC mutation is assumed to increase the symmetric division probability to 0.2 and bias it by a bias factor of 0.5. The mutation rate used here does not have any real basis and a high value is used in order to speed up the computation time. Our aim in performing these experiments is only to see the relative effects that symmetric division probability and differentiation probability have on the time for acquiring a second mutation and therefore actual values of the times are not important in these investigations.

**Table 4:** 

Effect of heterozygous APC mutation	Symmetric division probability	Bias factor for differentiation probability	Average number of iterations to the second mutation
No effect	0.1	1	6460
Increased symmetric division	0.2	1	6220
Increased biased symmetric division	0.2	0.5	3939

We see that increased symmetric division by itself does not has a significant effect on the time taken for the second mutation. When the symmetric division is biased in favor of stem cell progeny the time for appearance of the second mutation is reduced. Thus the hypothesis that heterozygous APC mutation results in *biased* symmetric division, is able to explain the increase of somatic mutation rates in FAP crypts.

In summary, the hypothesis that heterozygous APC mutation increases symmetric division of stem cells as well as biases the division in favor of stem cell progeny, results in the following effects:

1. FAP crypts have increased stem cell numbers and increased niche succession period.
2. Sporadic APC mutation has a high probability of getting fixed in the crypt by niche succession. On the other hand a mutation that increases proliferation rate increases the chance of extinction of the stem cell lineage.
3. Somatic mutation rates are increased in cells which have APC mutation.

Thus through getting dominance in the crypt, through increase of stem cell number and consequent crypt fission, and increased chances of replication errors, heterozygous APC mutation sets the stage for colorectal cancer. If the molecular mechanism by which the mutant APC effects the increase of biased symmetric division can be experimentally deciphered it can lead to drugs that could prevent the progression to cancer in people who have inherited or acquired a mutation in one of their APC alleles.
